# Mucosal urinary secretion revealing adenocarcinoma of urachus

**DOI:** 10.1016/j.amsu.2021.102335

**Published:** 2021-04-21

**Authors:** Issam Jandou, Adnan Ettanji, Ettaouil Mohammed, Amine Moataz, Dakir Mohammed, Adil Debbagh, Rachid Aboutaieb

**Affiliations:** a University Hospital Center Ibn Rochd Casablanca, Morocco; b Faculté de Médicine et de Pharmacie Casablanca, Morocco; c Laboratoire de Santé Sexuelle, Faculté de Médecine et de Pharmacie, Université Hassan II, Casablanca, Morocco

**Keywords:** Urachal adenocarcinoma, Bladder cancer, Hematuria, Urinary mucosal secretion

## Abstract

**Introduction:**

Urachus adenocarcinoma is an extremely rare malignant tumor characterized by its insidious evolution responsible for the delay in diagnosis. Several scientific works have tried to study the indication of adjuvant treatment, therefore the prognosis is still poor.

**Presentation of case:**

We report the case of a 50-year-old patient with no pathological history who consulted for an episode of intermittent urinary mucosal secretion aggravated by the appearance of macroscopic hematuria. Without other associated clinical signs. Imaging examinations revealed a mass at the expense of the upper wall of the bladder. The cystoscopy allowed us to visualize the mass and the biopsy. Histological study revealed an adenocarcinoma of urachus. The patient underwent surgical exeresis and adjuvant chemotherapy. The evolution was marked by a deterioration of the general condition despite adequate management.

**Discussion:**

Due to its topography, urachus cancer usually manifests as a bladder tumor, exceptionally as much as an anterior umbilical or extraperitoneal tumor. Few studies have been done on this neoplasm; however surgery still has a primary place in therapeutic management.

**Conclusion:**

The scarcity of cases of urachus cancer makes the publications scarce and the lack of multicenter clinical and randomized trials explains the disagreement about adjuvant treatments.

## Highlights

• Urachus cancer is a rare malignant tumor.
• The tomodensitometry is the reference examination which makes it possible to think of the diagnosis in front of a mass of urachus.
• Surgery remains the gold standard for therapeutic management.
• The prognosis is pejorative because of the aggressiveness of the tumor of the delayed diagnosis.

## 1 Introduction

Uracal cancer is a rare malignant tumor that develops at the expense of an embryonic remnant. It accounts for 0.17% of bladder tumors and 0.01% of adult cancers [1].

The clinical presentation remains insidious and variable ranging from a simple mucous urinary secretion to a giant abdominal mass. As a result of this silent clinical course, uracal adenocarcinoma still has a poor prognosis.

Imaging has an essential place in the diagnosis, the therapeutic strategy and the evolutionary follow-up. Surgery remains the benchmark for therapeutic care. Radiochemotherapy is still awaiting a randomized multicenter scientific evaluation and the advent of new, more effective molecules. Tumor markers such as CA125 play an important role in postoperative surveillance. The objective of our clinical case is to highlight one of the rare aspects of revelation of malignant uraque tumors and to discuss the possible methods of diagnosis and management. This work has been reported according to SCARE 2020 criteria [2].

## 2 Case presentation

A 50-year-old patient, mother of 3 children with no pathological history. She consulted for an episode of intermittent urinary mucosal secretion for 5 months worsened by the onset of macroscopic hematuria progressing for 1 month. Without other associated signs, all taking place in a context of conservation of the general condition. The abdominal examination, pelvic examinations as well as the somatic examination are unremarkable.

**Fig. 1:**
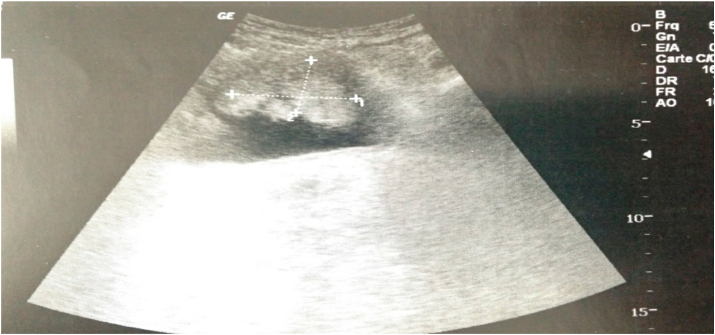
Ultrasound objectified echogenic tissue process of the anterior wall of the bladder.

The bladder ultrasound revealed an echogenic tissue process of the anterior bladder wall measuring 50 × 25mm (Fig. 1).

Cystoscopy performed under spine anesthesia uncovered a large bladder tumor at the expense of the anterior wall, sessile macroscopic appearance, fleshy and with a broad implantation base, suggesting an infiltrating tumor. The rest of the bladder lining was unremarkable.

The anatomopathological study after endoscopic bladder resection revealed a colloid mucous carcinomatous tumor proliferation. It shows, within large areas of mucus, the presence of isolated carcinomatous cells in the form of a signet ring or the form of small clumps. These extend over the entire mucosa with extension to the muscle layer. The pathologist concluded that there was a colloid mucous adenocarcinoma.

Laboratory workup revealed a hemoglobin level of 13.6 g/dl. Renal function is normal with blood urea at 0.25 g/l and serum creatinine at 7 mg/l. The tumor markers showed no abnormalities, the CA 125 at 19.2 U/ml and the ACE at 19.96 ng/ml.

**Fig. 2:**
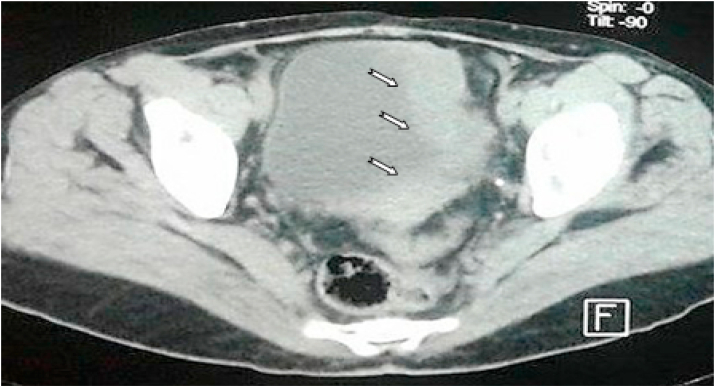
Abdomino-pelvic CT revealed bladder tumor process budding at the expense of the left lateral wall.

**Fig. 3:**
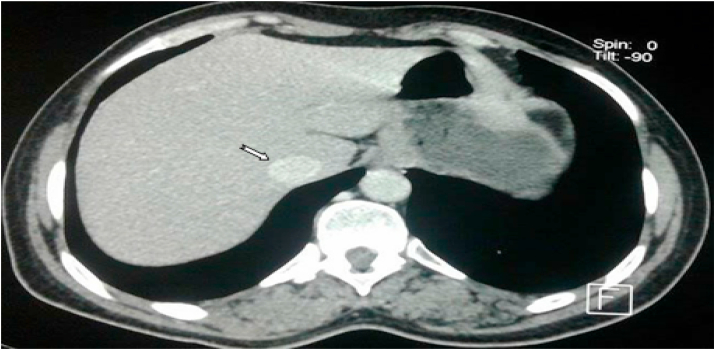
Abdomino-pelvic CT showed well-limited hepatic segment VI damage, discreetly enhancing after injection of PDC.

**Fig. 4:**
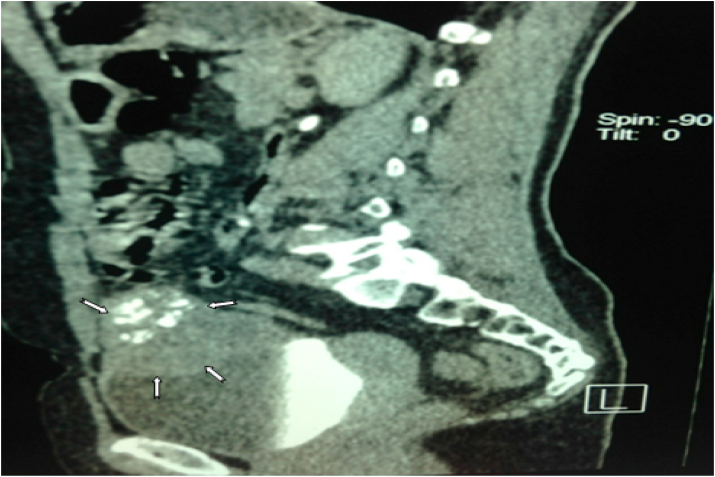
Abdomino-pelvic CT revealed a bladder tumor process budding at the expense of the left anterolateral wall with exophytic development and site of calcifications.

Computed tomography shows a bladder tumor process budding at the expense of the anterior and left lateral wall with exophytic development and site of calcifications. It measures approximately 72 × 48.6 × 73.2mm. It is also associated with an infiltration of neighboring fat. Also, the liver is of normal size. At the level of its segment, VI sits a hypodense nodular lesion measuring 14.5 × 14mm, discreetly enhancing after injection of PDC (Fig. 2; 3 and 4).

The patient underwent a rectosigmoidoscopy to look for a neighboring tumor that was without abnormality. After multidisciplinary consultation, it was decided to start with surgical treatment, then to refer the patient to the oncology department for adjuvant chemotherapy. The patient was operated on in our urology department.

Two weeks after confirmation of the diagnosis, the operative procedure was performed under general anesthesia after draping and bladder catheterization. It consisted of performing an anterior pelvectomy removing the bladder with urachus as well as the uterus, appendages and anterior wall of the vagina. The urinary diversion was a bilateral ureterostomy. Postoperative clinical and laboratory monitoring was without abnormalities.

The anatomopathological examination of the surgical specimen shows a resection specimen carrying the bladder and the uterus in one piece with the appendages in place, the right ureter is resected over 2cm, the left over 1.5cm; the outer surface of the bladder is multi-nodular in appearance. Microscopic examination noted the presence of a submucosal neoplasm at the level of the bladder dome and on the anterior surface, ulcerating in places the bladder mucosa and budding endoluminal. The neoplasm is shiny whitish in appearance, the site of extensive calcium changes. It largely infiltrates the muscular and serosa bladder remaining 1mm from the excisional surface and respecting the uterine wall. The right and left lymph node dissection each weighs 10g.

Histological examination shows that the neoplasm corresponds to a carcinomatous proliferation infiltrating the bladder wall from the outside to the inside with a maximum tumor volume located in the serosa and muscularis.

The proliferation is arranged in large pools of mucus cut into lobules by fibrous septa. Within these pools are found carcinomatous structures arranged in clusters and tubes or isolated cells taking a Signet ring appearance. The cells have abundant mucosecreting cytoplasm and atypical nuclei with abnormal mitoses. It is noted vascular emboli and peri-nervous sheaths. The proliferation comes into contact with the exeresis surface of the bladder serosa.

The bladder mucosa is free from carcinoma in situ lesions and foci of glandular metaplasia. The urethral and ureteral extremities are free.

The right ovary is infiltrated by the carcinomatous proliferation described above and the right tube, as well as the left appendix, do not present any particularities.

The pathologist concluded that there was a bladder localization of a mucous colloid adenocarcinoma with a signet ring cell component. Presence of vascular emboli and perinervous sheath. The margins of exeresis of the bladder serosa were tumor with right ovarian metastasis and absence of lymph node metastasis.

The immunohistochemical study shows a carcinomatous proliferation diffusely expressing CK7, ACE, and focal CK20, CDX2. The patient was subsequently referred to the oncology department where she received adjuvant chemotherapy such as Folfox based on oxaliplatin, folic acid, and 5-fluorouracil.

**Fig. 5:**
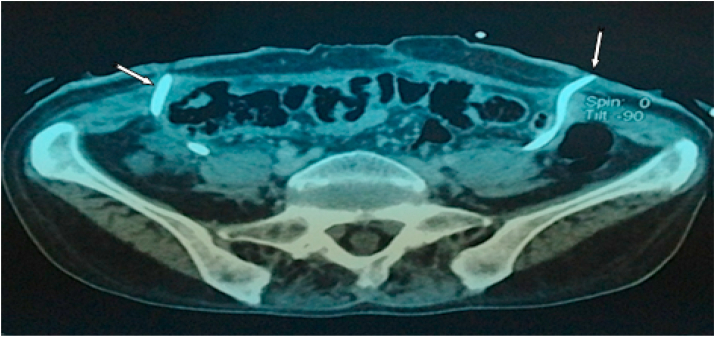
Thoraco-abdominal-pelvic CT scan showed Infiltration of pelvic fat with no detectable mass. Ureterostomies are connected to the abdominal wall.

She received 10 cures and presented side effects consisting of intense fatigue, nausea, vomiting, and transit disorders, all supported by symptomatic treatment. Control CT scan 1 month after surgery: shows pelvic fat infiltration without detectable mass, with persistence of hepatic lesion of segment VI (Fig. 5).

The evaluation computed tomography one year after treatment: shows an aspect of peritoneal carcinoma made up of a significant nodular infiltration of the pelvic fat, site of calcifications, supra, and retrohepatic.

**Fig. 6:**
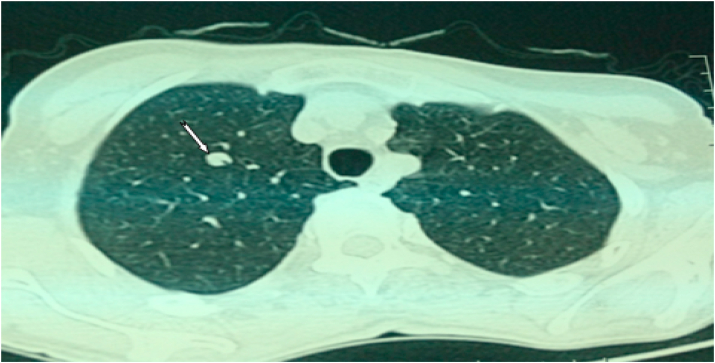
Thoraco-abdominal-pelvic CT scan revealed Right apical nodule.

**Fig. 7:**
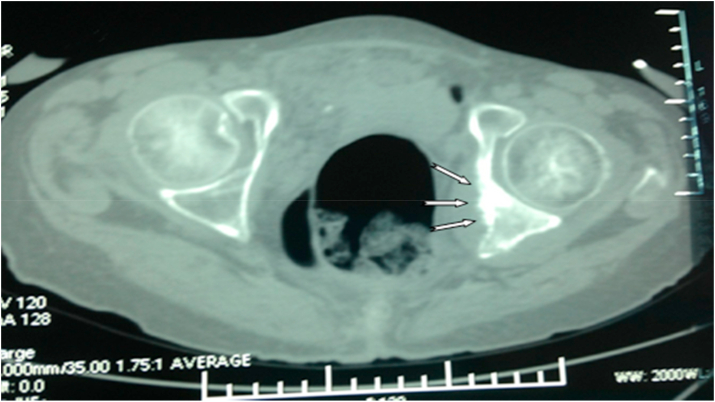
Abdomino-pelvic CT scan showing Bone lesion of the left acetabulum, with reaction of the periosteum burning grass.

Lung lesions formed by two nodules, right apical and middle lobar with non-septal postero-basal thickenings. Well, limited hepatic hypodense lesion of segment VI measuring 11 × 10mm. Mixed bone lesions, with periosteal reaction to grass fire of the left acetabulum and the right ischiopubic branch (Fig. 6, Fig. 7).

A computed tomography scan two years after treatment revealed the presence of three dense parenchymal pulmonary nodules with upper right lobed polylobed contours. The dysmorphic liver has several hypodense nodules, two in segment VI, one in segment II and four in segment IV. Lumpy infiltration of peritoneal fat with an effusion of moderate abundance. Mixed bone lesions with periosteal reaction to grass fire involving the two ischio-pubic branches and the left acetabulum with infiltration of the adjacent soft parts.

Over two years, the patient underwent regular clinical and paraclinical evaluation. Clinically, the patient weakened and became bedridden and cachectic (PS: 3), despite adequate vitamin-protein supplementation. Radiologically, the evolution was marked by an aggravation of the pre-existing lesions as well as the appearance of new pulmonary, hepatic, peritoneal, and bone lesions. The patient died two years after the start of treatment.

## 3 Discussion

The first citation of the urachal tumor in the literature was reported by the scientific work of Hue and Jacquin in 1863 [3]. Uraque cancer is a rare tumor with a particularly complex incidence to define, it is estimated at 1/5,000,000. The age of predilection for developing this neoplasm is between 40 and 60 years with a sex ratio ranging from 2 to 4 men for a woman according to several studies [4,5]. However, it represents 0.17% of all bladder tumors and 0.01% of adult cancers [6].

From an embryological point of view, the uraque and the dome of the bladder have the same endodermal origin while the rest of the bladder has a mesodermal origin. This origin has an impact on the choice and the therapeutic strategy [7]. The urachus is located in an anatomical doubling of the umbilico-vesical fascia, which limits the spread of cancers from the urachus to the parietal region [8].

Due to its topography, urachus cancer usually manifests as a bladder tumor, exceptionally as much as an anterior umbilical or extraperitoneal tumor [8].

Hematuria is a vital sign of urachal cancer. It is often associated with other functional or physical signs that are rarely isolated. Its abundance is variable (low, medium, or severe), it is generally terminal or total with terminal reinforcement [9].

Irritative signs are present in 50% of cases, attesting to compression or infiltration of the bladder wall of the urachus. Hypogastric pain is found in 20% of patients, which proves the non-specificity of this sign. The presence of an abdominal mass is encountered in 9–32% of cases, urinary mucous secretions, umbilical fistula, and distant metastases are rare but can also be revealing [[10], [11], [12]].

Punctiform hypogastric calcifications can be objectified by an abdominal x-ray without preparation. Intravenous urography (IVU) shows subtraction images at the level of the bladder dome at a late stage of development. IVU, retrograde cystography, and fistulography are no longer of interest after the advent of CT scans [13].

Abdominopelvic ultrasound may show bladder invasion and metastatic secondary locations. In 90% of cases, a cystoscopy coupled with an abdominal palpation can reveal a tumor of the anterior surface or the dome and make possible a transurethral biopsy [14].

Computed tomography is the gold standard, in specific ways the tumor is divided into an intravesical caudal part and a cystic, solid, or supra-bladder solidocystic part. The presence of calcifications which are often peripheral can also be central. It also determines the degree of bladder, lymph node, or metastatic invasion. It also guides the performance of scano-guided biopsies and the monitoring of local or remote recurrences after surgery [15]. Magnetic resonance imaging (MRI) in Uraquian tumors is more advantageous than CT. The extension assessment is thus more precise to the bladder, peri-vesical fat, the wall, to adjacent structures, and the lymph nodes [15,16].

The gold standard for surgery has long relied on omphalectomy, extensive resection of the urachus, anterior parietal peritoneum, posterior rectus fascia, as well as cystectomy and prostatectomy in men. In practice, performing a total cystectomy remains controversial because of the possibility of a 2cm excision margin at the level of the bladder section. An extemporaneous examination will be of great importance intraoperatively. However, partial cystectomy and omphalectomy are correlated with a five-year survival of the same order as that obtained after radical surgery with a higher local recurrence rate [7,17].

Uraque cancers are not very radiosensitive, nevertheless an adjuvant postoperative irradiation of the tumor bed or in inoperable cases, as well as to treat local pain and direct tumor compressions or by metastases.

According to several studies, the benefit of chemotherapy remains ineffective. One job that compared the cisplatin and 5FU-based protocol with abstention found a similarity in overall survival. An elevated level of CA125 is a useful marker for postoperative follow-up which may warrant additional chemotherapy [18].

## 4 Conclusion

The scarcity of cases of urachus cancer makes the publications scarce. The absence of a specific clinical sign leads to a delay in diagnosis, which is responsible for the poor prognosis of urachal adenocarcinoma. Ultrasound can detect this tumor at an earlier stage. TMD and MRI have a central place in diagnosis, treatment, and monitoring. The therapeutic strategy consists of a partial or total resection of the bladder including the urachus and the umbilicus. The lack of multicenter clinical and randomized trials explains the disagreement about adjuvant treatments.

## Ethical approval

The study committee of the jura sud hospital center approves the favorable opinion to publish this work.

## Funding

We have any financial sources for our research.

## Author contribution

Dr. EJ, Dr. IJ, and Dr. CB analysed and performed the literature research, Pr. HL performed the examination and performed the scientific validation of the manuscript. Issam Jandou was the major contributors to the writing of the manuscript. All authors read and approved the manuscript.

## Registration of research

Name of the registry:

Unique Identifying number or registration ID:

Hyperlink to your specific registration (must be publicly accessible and will be checked):

## Guarantor

Dr. Jandou issam.

## Consent

The consent to publish this information was obtained from study participants. We confirm that written proof of consent to publish study participants are available when requested and at any time.

## Declaration of competing interest

All authors disclose any conflicts of interest.
